# “*Flower Power*”: Controlled Inhalation of THC-Predominant Cannabis Flos Improves Health-Related Quality of Life and Symptoms of Chronic Pain and Anxiety in Eligible UK Patients

**DOI:** 10.3390/biomedicines10102576

**Published:** 2022-10-14

**Authors:** Guillermo Moreno-Sanz, Alvaro Madiedo, Michael Lynskey, Matthew R. D. Brown

**Affiliations:** 1 Khiron Life Sciences Spain, 28001 Madrid, Spain; 2 Khiron Life Sciences, Bogotá 110221, Colombia; 3 Drug Sciences, London SW7 2BX, UK; 4 Zerenia Clinics, London SW1X 9AE, UK; 5 The Royal Marsden Hospital, London SW3 6JJ, UK

**Keywords:** cannabis, chronic pain, anxiety, inhalation, tetrahydrocannabinol, HRQoL

## Abstract

In November 2018, the UK’s Home Office established a legal route for eligible patients to be prescribed cannabis-based products for medicinal use in humans (CBPMs) as unlicensed medicines. These include liquid cannabis extracts for oral administration (“oils”) and dried flowers for inhalation (“flos”). Smoking of CBPMs is expressly prohibited. To date, THC-predominant cannabis flowers remain the most prescribed CBPMs in project Twenty21 (T21), the first multi-center, prospective, observational UK cannabis patient registry. This observational, prospective data review analyzes patient-reported outcome measures (PROMS) collected by T21 associated with the inhalation of KHIRON 20/1, the most prescribed CBPM in the project. PROMS collected at baseline and at subsequent 3-month follow-up included health-related quality of life (HRQoL), general mood, and sleep. Condition-specific measures of illness severity were performed with the Brief Pain Inventory Short Form (BPI-SF) and the Generalized Anxiety Disorder 7-Item Scale (GAD-7). Participants (N = 344) were mostly males (77.6%, average age = 38.3) diagnosed mainly with chronic pain (50.9%) and anxiety-related disorders (25.3%). Inhalation of KHIRON 20/1 was associated with a marked increase in self-reported HRQoL, general mood, and sleep (N = 344; *p* < 0.001). Condition-specific assessments showed significant improvements in pain severity (T = 6.67; *p* < 0.001) and interference (T = 7.19; *p* < 0.001) in patients using KHIRON 20/1 for chronic pain (N = 174). Similar results were found for patients diagnosed with anxiety-related disorders (N = 107; T = 12.9; *p* < 0.001). Our results indicate that controlled inhalation of pharmaceutical grade, THC-predominant cannabis flos is associated with a significant improvement in patient-reported pain scores, mood, anxiety, sleep disturbances and overall HRQoL in a treatment-resistant clinical population.

## 1. Introduction

Cannabis was (re) introduced into British medical practice in the early 1840’s by Irish physician Dr. William O’Shaughnessy, an army surgeon serving in Calcutta, India [1]. In the Victorian period, cannabis was widely used for a variety of ailments, including muscle spasms, menstrual cramps, rheumatism, the convulsions of tetanus, rabies, and epilepsy, and as a sedative. Cannabis extracts were typically administered orally in the form of an alcoholic tincture and were commonly incorporated in proprietary medicines [2]. With the introduction of synthetic drugs, herbal remedies were increasingly viewed as unpredictable and many of them, including cannabis extracts and tinctures, were removed from the British Pharmacopoeia of 1932 but retained in the British Pharmaceutical Codex of 1949. Under the Dangerous Drugs Act 1964, which implemented the 1961 UN Single Convention on Narcotic Drugs in the United Kingdom, the prescription of cannabis tinctures continued to be permitted due to a “license of right” received under the Medicines Act 1968. However, this license of right was subsequently not renewed, and the original Misuse of Drugs Regulations of 1973 listed cannabis, cannabis resin, cannabinol and its derivatives in Schedule 4 (now Schedule 1) completely prohibiting medical use [2]. In November 2018, the UK’s Home Office (re) established a legal route for the prescription of cannabis-based products for medicinal use in humans (CBPMs) through the amendment of both the Misuse of Drugs Regulations 2001 and Misuse of Drugs Order 2015, rescheduling CBPMs as Schedule 2 drugs [3]. CBPMs remain strictly regulated and include both cannabis extracts for oral administration (“oils”) and dried cannabis flowers for inhalation (“flos”). These products may only be prescribed by a specialist medical practitioner as “special” or “bespoke” medications following processes common to all unlicensed medications.

Whilst smoking of cannabis and CBPMs is expressly prohibited in the legislation, cannabis flos remains the most popular cannabis galenic formulation in the UK, a situation similar to that which occurs in other jurisdictions with established medicinal-cannabis access schemes, such as Germany, Canada, and Israel [4]. Qualitative research studies have shown that patients using cannabis for therapeutic purposes tend to choose the inhalation of flos as their preferred method of administration, as it provides a greater control over dosage and speed of onset, as well as a more robust relief of symptoms compared to the oral route [5]. Additionally, the development of vaporizers and inhalers for flos, some of which have attained certification as medical devices, affords patients greater control over administration and dosing of the pharmacologically active molecules present in cannabis, namely cannabinoids Δ9-tetrahydrocannabinol (THC) and cannabidiol (CBD), limiting the occurrence of side effects related to the central nervous system and the inhalation of toxic by-products of combustion [6].

Oral THC has been clinically approved for the treatment of several health conditions, such as chemotherapy-induced nausea and vomiting, wasting syndrome associated with AIDS and cancer, and spasticity in patients with multiple sclerosis, and its ability to treat other neurological conditions is under investigation [7]. A large body of scientific literature indicates that inhalation of chemotype I (THC-predominant) cannabis flos can mitigate symptoms associated with chronic pain, increase relaxation, and facilitate resilience to cope with disability. A series of small placebo-controlled, randomized control trials (RCT) conducted with cannabis flos have shown that this therapy option is efficacious and safe at treating neuropathic pain, whilst also improving mood and daily functioning to a similar extent during treatment periods [8,9,10,11,12,13]. Analogous results were observed in a placebo-controlled crossover trials investigating patients with multiple sclerosis, in which perception of pain was a secondary outcome [14], or patients with chronic pain of varying etiology [15]. In addition to these RCTs, numerous observational studies contribute to a robust body of real-world evidence (RWE) which suggests that the inhalation of chemotype I cannabis flos could effectively ameliorate other types of chronic pain including pelvic pain [16], migraines [17], or fibromyalgia [18], as well as markedly improve various traumatic psychiatric conditions such as stress, anxiety, or depression [19,20,21].

A recent single-center, observational study explored the clinical outcomes associated with the use of CBPMs in British patients diagnosed with chronic pain, a condition that affects approximately 28 million people in the UK with an estimated direct and indirect cost of £21.2 billion [22]. To minimize the variability in the formulation, participants were prescribed one single oral cannabis extract normalized in medium-chain triglycerides (MCT) oil. Product composition and route of administration are typically difficult to control for and a frequent confounding factor in observational studies. Authors reported significant improvements in health-related quality of life, pain interference and sleep quality, accompanied by a 30% incidence of side effects of mild or moderate intensity [22]. Following a similar rationale and experimental design, in the present work we aimed at investigating the efficacy and safety of the inhalation of THC-predominant cannabis flowers on a treatment-resistant cohort of patients enrolled in Project Twenty21 (T21), the first multi-center registry of patients receiving bespoke CBPMs in the UK [23,24]. We analyzed clinical outcome measures, collected prospectively through validated questionnaires [25], reported by patients receiving treatment with KHIRON 20/1, the most frequently prescribed chemotype I cannabis flower in T21.

## 2. Materials and Methods

### 2.1. Design

We analyzed clinical data collected prospectively between August 2020 and June 2022 to investigate the clinical outcomes associated with the inhalation of THC-predominant flos for therapeutic purposes in a legal and medically supervised setting. Participants were patients registered in Project Twenty21 (T21), the first UK multi-center registry seeking to develop a body of real-world evidence (RWE) to inform on the effectiveness and safety of medical cannabis. Full information relating to T21 procedures is outlined elsewhere [23,25]. In brief, patients receiving CBPMs for a variety of conditions are entered into the registry by invitation and monitored for data collection as part of their standard of care. According to UK regulations, individuals must have an established diagnosis and have failed to respond to at least two treatment options to legally receive CBPMs. Patients provided consent (following Good Clinical Practice guidelines) to the collection of their medical history, past and current treatments, plus a series of symptomatic assessments based on standardized and comprehensively validated self-report questionnaires. Prescribing physicians partnering with T21 use a product formulary that includes a wide range of CBPMs including oral extracts and flos of differing CBD and THC ratios. To date, THC-predominant flos remains the most prescribed CBPM in the project [23]. To reduce the inherent variability associated with the chemical composition of cannabis dried flowers, we decided to include in our data review only those patients receiving at their initial appointment a prescription for KHIRON 20/1, the most frequently prescribed THC-predominant flos in T21. Additional inclusion criterion was that participants had completed health-related quality of life (HRQoL) questionnaires both at the initial appointment (baseline) and at the subsequent 3-month follow up.

### 2.2. Drugs

KHIRON 20/1 (Pharmadrug Production GmbH, Rostock, Germany) is a chemotype 1 cannabis variety which contains 20% (*w*/*w*) of THC and less than 1% (*w*/*w*) of CBD in dried weigh. This variety is also referred to by the breeder’s name *Hindu Kush* and is classified as an indica-type plant. Indica/sativa terminology relates to structural and botanical features of the cannabis plant and, contrary to what commonly misconstrued, does not provide robust information on the chemical composition nor on the pharmacological characteristics of the flos [26]. The batches of KHIRON 20/1 flos prescribed to T21 participants were produced in full compliance with good manufacturing practices (GMP) requirements and to the standards established in the German monograph for cannabis flos [27].

### 2.3. CBPM Administration Protocol

**Figure 1:**
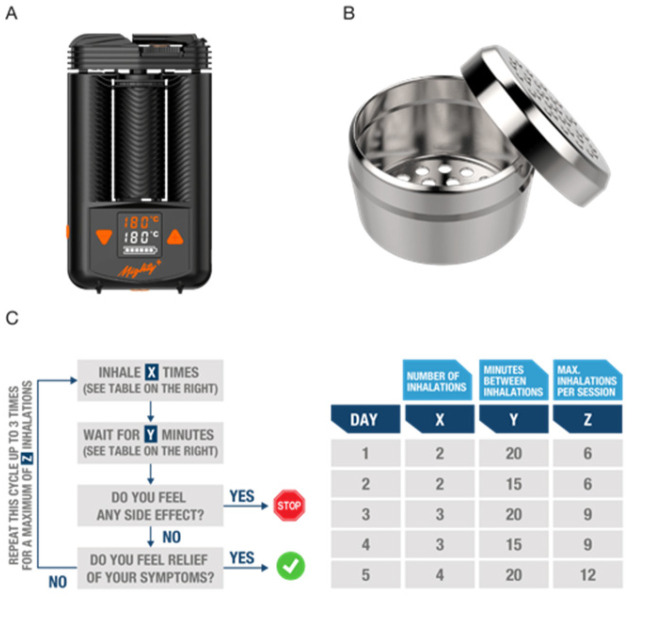
Proposed protocol to initiate naïve patients and prescribing doctors safely into the inhalation of THC-predominant cannabis flos. (**A**) The herbal vaporizer mightly medic is powered by rechargeable batteries and attained EU-mark as a medical device; (**B**) Pharma-grade aluminum dosing capsules holding up to 0.25 g of grinded cannabis flos can be loaded into the mighty heating unit. (**C**) Flowchart depicting a proposed 5-day familiarization plan for naïve users, with daily increases in number and frequency of inhalations to minimize the risk of CNS-related side effects.

The UK´s Misuse of Drugs Regulations 2018 explicitly prohibits smoking of cannabis and CBPMs, therefore, an herbal vaporizer/inhaler is required for the therapeutic administration of cannabis flos. Currently, there are two vaporizers that have attained the EU certification of medical devices for the inhalation of cannabis flowers, both manufactured by the German company Storz&Bickel: the Volcano medic, a tabletop model [28], and the battery-operated, handheld device Mighty Medic [29]. Although most clinical research on vaporizing medicinal cannabis has been performed using the Volcano device, the majority of T21 participants typically prefer a handheld device, such as the Mighty Medic (Figure 1A), both for convenience and economic reasons. Owing to the more rapid effect onset, inhalation allows the experienced patient to easily titrate the dosage to maximize therapeutic benefit and minimize side effects typically related to overt THC-related psychoactivity, by controlling the number, duration, and frequency of inhalations. Figure 1C illustrates the dosing protocol we developed to guide T21 prescribers and cannabis-naïve participants through the process of personalizing cannabis inhalation depending on the needs of each individual patient. In brief, to vaporize THC-predominant flowers, patients are advised to:

1. Fill the Mighty Medic dosing capsules with grounded cannabis flos (Figure 1B). Although the maximum capacity per capsule is 250 mg, dosing is based in both the number and frequency of inhalations rather than the absolute amount of herbal material loaded into the device. This allows an experienced patient to have more control over administration and dosing while, at the same time, adjust and standardize the amount of cannabis flos used to optimize cost–benefit.
2. Turn on the device and set the temperature to 180 °C (Figure 1A). At this temperature, vapor will be composed mainly of steam, most volatile terpenes (e.g., limonene, pinene), and small amounts of THC (boiling point 157 °C) which will start decarboxylating.
3. Once the target temperature is reached, patients are instructed to inhale and exhale naturally. Vapor should not be held in the lungs longer than during regular breathing. The first inhalation is typically less effective since it serves to “prime” the device and warm up the herbal material.
4. After inhaling the indicated number of times (see Figure 1C), patients are advised to wait for 15–20 min and observe for side effects (such as dizziness, tachycardia, nausea, disorientation, euphoria, etc.). After this period, and in absence of side effects, patients can repeat the cycle if symptomatic control has not been achieved, increasing the temperature by 10 °C (Figure 1C).
5. At 190 °C and 200 °C the vapor may feel dryer and less fragrant but will be more concentrated in cannabinoids [30]. Vaporization of cannabinoids continues at high temperatures even if vapor is not visible when exhaling, due to the exhaustion of water in the herbal material.
6. The goal for this 5-day initiation protocol is to provide the prescribing doctor with clear administration instructions to share with patients so that they can experiment with the device and familiarize themselves safely with cannabis inhalation.

### 2.4. Patient-Reported Outcome Measures (PROMS)

PROMS questionnaires are completed by T21 participants both at baseline/treatment entry and then every 3 months at scheduled follow-ups. The following questionnaires were employed to capture outcome measures that were either common for all participants (HRQoL, Mood and Sleep) or specific for each diagnosed condition (chronic painful conditions or anxiety-related disorders).

#### 2.4.1. Health-Related Quality of Life

The EuroQol 5 Dimensions (EQ-5D-5L) is a widely used, validated, and reliable tool to assess the quality of life of patients in many disease areas through evaluating the severity of each of 5 dimensions (mobility, self-care, usual activities, pain/discomfort, and anxiety/depression) [31]. Two measures of HRQoL were considered:

1. The visual analog score (VAS) of general health (0–100) was interpreted as a patient-reported measure of general health.
2. The sum of ratings for the five dimensions of the EuroQol (5–25) was interpreted as patient-reported measure of HRQoL.

#### 2.4.2. Mood/Depression

The Patient Health Questionnaire (PHQ-9) is a reliable and valid measure of depression severity, which is comprised by a 9-item, self-rated instrument previously validated in general populations, medical populations, and psychiatric samples [32]. Scoring ranges from 0 to 27.

#### 2.4.3. Sleep Disturbances

Quality of sleep was assessed by using four items adapted from the widely used Pittsburgh Sleep Quality Index [33]. Scoring ranges from 4 to 20.

#### 2.4.4. Chronic Pain

Participants diagnosed with chronic pain were asked to complete the Brief Pain Inventory Short Form (BPI-SF). The BPI-SF is validated in patients with both cancer and non-cancer pain and is one of the most used measurement tools for evaluating clinical pain, including both pain severity and the interference of pain on feelings and function [34]. Therefore, items from this scale were used to assess two distinct dimensions of pain: (i) severity of pain; and (ii) the extent to which pain interferes with daily activity. Patients scored both dimensions on a 0–10 scale.

#### 2.4.5. Anxiety

Participants diagnosed with anxiety-related disorders were asked to complete the Generalized Anxiety Disorder 7-Item Scale (GAD-7). The GAD-7 is one of the most frequently used, validated, self-reported questionnaires clinically employed to screen for, diagnose, and assess the severity of generalized anxiety disorder [35]. Each item is scored 0–3 for a composed total range 0–21.

### 2.5. Statistical Analysis

Demographics are expressed either as percentage or as the mean ± standard deviation. Results of PROMS analysis are represented in box and whisker graphs, which indicate upper and lower extreme values, median, upper quartile, and lower quartile. Statistical analyses were performed by either student’s t (comparisons of means at t = 0 and t = 3) or one-way ANOVA (comparisons of means at t = 0, t = 3 and t = 6) followed by Friedman non-parametric test and pair-wise comparisons (Durbin-Conover) using the Jamovi free software V2.2.2 (San Francisco, CA, USA). Post hoc analyses were considered statistically significant if *p* < 0.05.

## 3. Results

### 3.1. Participants

A total of 344 patients registered in T21 satisfied the inclusion criteria of (i) having PROMS questionnaires correctly recorded at the initial appointment (t = 0) and, at the least, at the 3-month follow up (t = 3), and (ii) receiving a prescription for KHIRON 20/1 at t = 0. Of those, 140 participants had also reported PROMS at the 6-month follow up (t = 6). Participants enrolled in T21 between August 2020 and June 2022. Demographics and clinical characteristics of the patient cohort are depicted in Table 1. Coherent with the overall patient population of T21, three out of four participants were adult males (77.6%), with an average age of 38.4 ± 10.4 years old. A majority of them were diagnosed with a chronic painful condition (50.8%) or an anxiety-related disorder (25.3%). Other minor qualifying diagnosis were Attention Deficit Hyperactivity Disorder (ADHD) (6.98%), Post Traumatic Stress Disorder (PTSD) (6.1%) or insomnia (2.9%). As required by law, all patients had trialed at the least two standard therapeutic options to treat their condition before accessing medicinal cannabis. Of note, only 16 participants were naïve to cannabis when commencing the T21 process. A vast majority of patients (95.6%) had previously utilized illicitly acquired cannabis, and 3 out of 4 of those consuming cannabis did so with the intention of treating their primary diagnosed condition. Most participants elected to administer CBPM once a day (58.7%).

### 3.2. General Health Outcome Measures

#### 3.2.1. Health Related Quality of Life (HRQoL)

**Figure 2:**
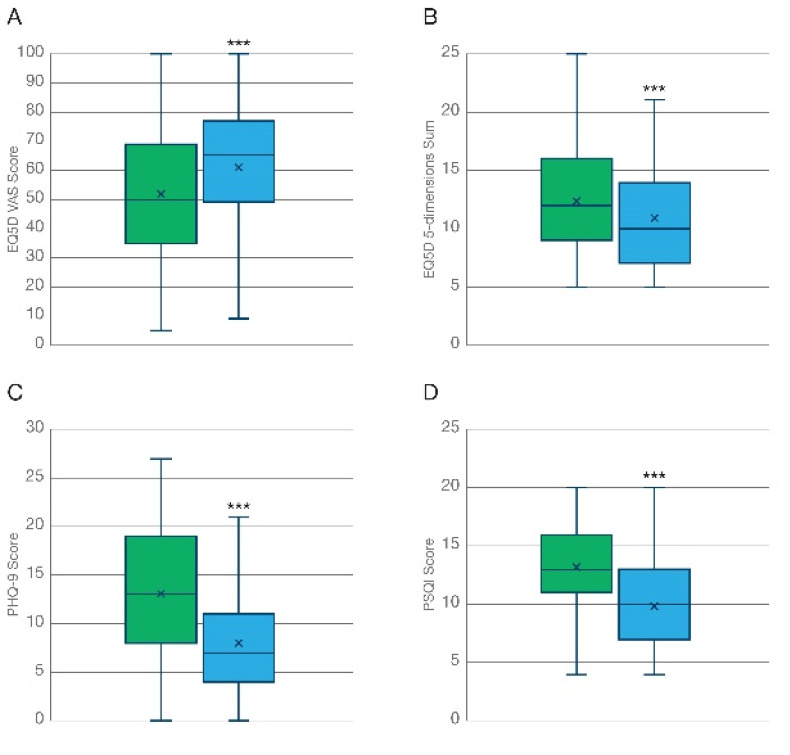
Improvement of general health outcome measures at the 3-month follow-up. Analysis of PROMS shows how the inhalation of KHIRON 20/1 was associated with a marked improvement in self-reported health related QoL (N = 344) measured as (**A**) the scoring of the VAS of the EQ5D, and (**B**) the sum of the 5 dimensions of the EQ5D questionnaire. (**C**) General mood/ clinical depression assessed with the PHQ-9 scale (N = 339) was markedly improved. This effect was mainly driven by patients diagnosed with anxiety-related disorders (Table 2). (**D**) Quality of sleep assessed with the Pittsburgh Sleep Quality Index (N = 344) was improved by the treatment with inhaled chemotype 1 cannabis flos. *** *p* < 0.001.

**Figure 3:**
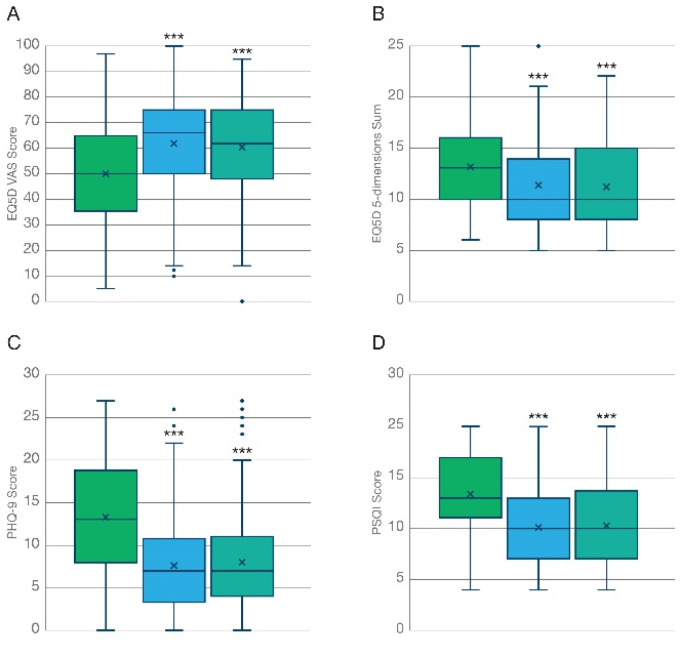
Comparative improvement of general health outcome measures at 3- and 6-month follow-up. Analysis of outcome measures reported by patients that completed the validated questionnaires both at 3- and at 6-month follow-up visits (N = 140). Improvements associated with the inhalation of KHIRON 20/1 in (**A**,**B**) HRQoL, (**C**) General mood/ clinical depression, and (**D**) quality of sleep were maximal at 3 months and maintained, although not further increased, at the 6-month follow-up. *** *p* < 0.001.

Inhalation of THC-predominant cannabis flos was associated with a marked improvement both in general health and in health-related quality of life (HRQoL) after 3 months, expressed as the VAS score (Figure 2A, T = 8.80; *p* < 0.001) and the sum of ratings for the 5 dimensions of the EuroQol (Figure 2B T = 10.3; *p* < 0.001), respectively. A similar degree of improvement was reported by participants at the 6-month follow up (Figure 3A,B), which is suggestive of (i) the maximal effect of the treatment being already achieved at the 3-month timepoint which was maintained but not further improved at 6 months, and (ii) no overt tolerance to the treatment developing after 6 months of daily administration. As shown in Table 2, participants diagnosed with chronic pain reported lower baseline levels of HRQoL compared to those diagnosed with anxiety disorders. However, no significant differences in the degree of improvement captured by the EQ-5D were found between these two groups at the 3-month follow up. On the contrary, patients diagnosed with anxiety-related disorders did report a larger improvement in general health compared to chronic pain patients (mean difference 12.4 vs. 6.98; *p* < 0.05), as captured by the VAS of the EuroQoL questionnaire.

#### 3.2.2. General Mood/Depression

Participants reported an improved overall mood associated with the treatment at the 3-month follow-up (Figure 2C, N = 339; T = 15.3; *p* < 0.001), which was maintained up to 6 months (Figure 3C, N = 136; Χ^2^ = 94.0; *p* < 0.001) as indicated by a significant reduction in the PHQ-9 questionnaire scoring. As shown in Table 2, the observed effect was strongly influenced by participants diagnosed with anxiety-related disorders, who reported slightly poorer baseline levels of mood/depression (13.72 vs. 12.78) and a significantly larger average improvement (7.14 vs. 3.36; T= −5.18; *p* < 0.01) in the PHQ-9 scale compared to those participants diagnosed with chronic pain after 3 months.

#### 3.2.3. Sleep Quality

Sleep deprivation is one of the most common comorbidities associated with chronic illness [36]. Quality of sleep, assessed by the Pittsburg sleep quality index (PSQI), was improved following the inhalation of THC-predominant cannabis flowers after 3 months (Figure 2D, N = 344; T = 14.5; *p* < 0.001). This effect was maintained, but not further increased, at the 6-month follow up (Figure 3D, N = 140; Χ^2^ = 74.9; *p* < 0.001). Participants diagnosed with anxiety disorders and chronic pain conditions reported similar basal levels (12.31 vs. 13.51) and no significant differences were found among the average improvement (3.15 vs. 2.72) in the PSQI scores of the two sub-populations (Table 2).

### 3.3. Indication-Specific Outcome Measures

Besides general outcome measures, which were collected for all patients, T21 participants are asked to complete health questionnaires specific to their primary indication. Here, we report only results from the main two health conditions, which included more than 85% of all participants (Table 1). Results from other less frequent indications, such as ADHD (N = 24) and PTSD (N = 21), will be disclosed in a separated data review once adequate statistical powering is achieved. Participants diagnosed with a chronic painful condition (N = 174) completed the Brief Pain Inventory-Short Form, a 9-item questionnaire used to evaluate (i) the severity of a patient’s pain and (ii) the impact of this pain on the patient’s daily functioning. Patients diagnosed with anxiety-related disorders or other mental health issues concomitant with anxiety (N = 107) completed the Generalized Anxiety Disorder (GAD-7), which total score for the seven items ranges from 0 to 21.

#### 3.3.1. Pain Severity

**Figure 4:**
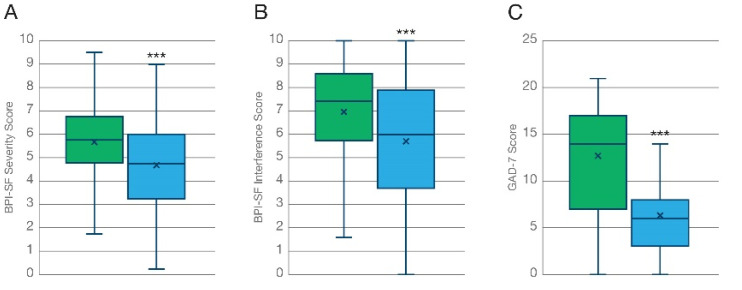
Improvement of indication-specific outcome measures at the 3-month follow-up. Analysis of PROMS shows how the inhalation of KHIRON 20/1 was associated with a marked improvement in self-reported (**A**) pain severity and (**B**) pain interference in patients diagnosed with chronic painful conditions, measured with the Brief Pain Inventory-short form (N = 174). (**C**) Generalized anxiety measured with the GAD-7 questionnaire was markedly decrease after 3 months of treatment with cannabis flos KHIRON 20/1 (N = 107). *** *p* < 0.001.

Participants reported a 16.2% reduction in pain severity (Figure 4A, N = 174; T = 6.67; *p* < 0.001) from an average baseline value of 5.63 to a mean value of 4.72 at the 3-month follow up, associated with the treatment.

#### 3.3.2. Pain Interference

Recovery of daily functioning and restoring “their old self” is one of the most recurrent features that chronic, self-medicating, patients associate to their therapeutic use of cannabis. Participants reported a 18.4% reduction in pain interference with their daily activities associated with the treatment from an average baseline value of 6.97 to a mean value of 5.69 at the 3-month follow up (Figure 4B, N = 174; T = 7.19; *p* < 0.001).

#### 3.3.3. Generalized Anxiety Disorder

Participants who completed the GAD-7 questionnaire reported a 50.7% reduction in anxiety symptoms (Figure 4C, N = 107; T = 12.9; *p* < 0.001) from an average baseline value of 12.7 to a mean value of 6.28 at the 3-month follow up.

### 3.4. Adverse Effects

T21 participants were also encouraged to report any adverse side effects that they considered associated with the treatment with CBPMs. Inhalation of THC-predominant flower was found in general to be safe. Only two participants diagnosed with chronic pain reported minor adverse side effects associated with KHIRON 20/1 from those available in the list: (i) a 42-year-old male with previous experience with cannabis who medicated several times a day reported suffering a “mild headache” which remitted after 1–2 h; and (ii) a 32-year-old female reported suffering “memory loss”. This adverse effect was described by the patient as “transient” and “not relevant”. Of note, this participant was among the 15 patients (4.4% of total cohort) that were naïve to cannabis prior to enrolling in project T21.

## 4. Discussion

The presented work investigates the ability of inhaled THC-predominant (chemotype-1) cannabis flos to improve health-related quality of life (HRQoL) and mitigate symptomatology in a treatment-resistant population of patients diagnosed with chronic painful conditions and anxiety-related disorders. Our results indicate that sustained inhalation of cannabis flos KHIRON 20/1 was associated with a robust and long-lasting improvement in HRQoL, mood and quality of sleep. The pharmacokinetics of orally ingested cannabinoids typically display erratic intestinal absorption, high inter- and intra-individual variability, extensive hepatic metabolism, and a delayed onset of effects between 90 and 120 min. In contrast, vaporized cannabinoids are rapidly and reliably absorbed into the bloodstream, achieving peak concentrations in blood generally in under 10 min [37]. These differences in pharmacokinetic properties afford patients a greater degree of control over dosage and speed of onset. Accordingly, both quantitative and qualitative research report on the ability of inhaled cannabis flos to quickly relieve symptoms of depression, stress, and anxiety. Patients describe the bodily sensation of cannabis inhalation as a “sigh of relief”, which leads to a state of relaxation promoting a reduction in pain sensation and, subsequently, improved sleep, motility, mood and acceptance [5,38]. For this reason, inhalation of cannabis flos is typically recommended as a rescue medication for acute or “breakthrough” symptoms [39]. However, our results showed sustained reduction in pain severity and interference of chronic pain with daily activities after 3 months of daily administration of KHIRON 20/1, which was maintained at the 6-month follow-up.

This finding is coherent with recent prospective observational studies investigating medical outcomes in chronic pain patients combining different formulations of medicinal cannabis, which also reported significantly lower levels of pain severity and pain interference, improved mood, sleep duration and sleep quality, and overall quality of life at 3 months compared to baseline [40,41]. Besides the 3-month follow up, Wang and collaborators incorporated ecological momentary assessment (EMA) to measure real-time health outcomes once daily for one week before (baseline) and for up to three weeks immediately after starting the treatment. Authors reported a significant reduction in real-time pain intensity (16.5-point reduction in a 0–100 VAS) and anxiety, longer sleep duration and better sleep quality in the first 3 weeks of treatment [40]. In similar studies, chronic pain patients treated exclusively with oral CBPMs also showed maximal improvement 3 months after the treatment initiation, which was sustained for over 6 months [22,42], suggesting that tolerance to the beneficial effects of cannabinoid therapy does not commonly occur. This observation is further supported by results from RCTs leading towards the clinical approval and commercialization of Sativex, in which MS patients showed sustained improvements in pain for more than 12 months without developing tolerance [43].

It is notable that greater than 95% of patients included in our data review were using cannabis illegally to treat their conditions at baseline and yet, we found a marked improvement in all PROMS analyzed. We interpret this finding to indicate that the administration of cannabis flowers in a clinical environment, under the supervision of a trained healthcare provider, further improves the clinical outcomes associated with legally prescribed CBPMs when compared to chronic patients self-medicating with illicit cannabis. This interpretation is further supported by similar findings from different jurisdictions where legally protected access to medical cannabis had recently become available [44,45]. The effect of such regulatory changes may have a greater impact in those experiencing anxiety-related disorders as it eliminates several major concerns for these patients, such as product availability, product reproducibility and the fear of potential legal consequences [5]. Our results indicate indeed that the largest clinical improvements associated with the inhalation of THC-predominant cannabis flos were reported by patients with a primary indication of generalized or social anxiety. First, we found a robust reduction in the GAD-7 scoring, from a baseline value of 12.7 to a value of 6.28 at the 3-month follow up. This remarkable result contrast with those reported by a Canadian group who applied the GAD-7 scale to a large cohort of adults authorized to use cannabis between 2014 and 2019. Although a statistically significant decrease in GAD-7 scoring was noted (from 9.11 to 9.04), it did not meet the threshold to be considered clinically significant [46]. In contrast, participants in our cohort diagnosed with generalized anxiety displayed higher baseline levels of moderate-to-severe anxieties, which could be potentially exacerbated by their illicit use of cannabis. Second, our result show that the cohort of patients diagnosed with anxiety-related disorders had a significantly larger contribution to the improvement in mood captured by the PHQ-9 scale, a measure of clinical depression. It could be postulated that the overall anxiolytic effect of whole flower CBPM could result from the combination of a rapid pharmacological activation of central type-1 cannabinoid (CB1) receptors together with the reassurance of pharmaceutical quality CBPM, legally prescribed by a clinician.

Functional imaging studies in humans have shown a correlation between THC-mediated analgesia and a reduction in neural connectivity between the anterior cingulate cortex (ACC) and cortical areas involved in pain processing, the dorsolateral prefrontal cortex in particular, which are two key brain regions for the modulation of cognitive and emotional inputs [47,48]. Accordingly, results from human lab experiments suggest that THC prevents the onset of pain sensation by slightly increasing pain threshold but does not effectively reduce the perceived intensity of experimental pain [49]. Instead, THC seems to influence affective processing, thus making pain sensation less unpleasant and more tolerable, which resonates with qualitative assessments made by patients treated with CBPMs [38]. Available evidence also suggests that inhaled THC can potentiate the extinction of fearful and aversive memories in humans and reduce anxiety responses without eliciting psychotic effects [50], although it remains unclear if this effect is mediated by the activation CB1 receptors in the same brain regions. However, significantly increased circuit coupling between the ACC and the amygdala has been described during the processing of fearful stimuli in anxious (but not in healthy) individuals, which also correlated positively with self-reported symptoms of anxiety [51]. The key regulatory role of CB1 receptors in the amygdala, activated by endogenously produced anandamide on fear processing and aversive memory extinction has also been characterized both in preclinical and clinical studies [52,53]. Taken together, this evidence highlights the role of the ACC as a critical mediator in the analgesic and anxiolytic actions of THC, which could also explain why frequent and transient activation of central CB1 receptors could lead to sustained improvement in the emotional processing and a reduction in negative affect and physical symptoms associated with chronic illnesses [19].

The occurrence of adverse side effects experienced by participants was relatively rare, likely because most participants (95.6%) had previous experience with cannabis inhalation. In fact, the one patient reporting transient, mild, memory loss was naïve to cannabis. Adverse CNS-related side effects following cannabis inhalation are typically related to the dose of THC [54]. To counter this we have detailed an administration protocol to guide naïve patients and prescribing doctors following the mantra of “start low and go slow” [39], and based on the number and frequency of inhalations as opposed to the total amount of herbal cannabis loaded in the vaporizer. Pharmacokinetic studies on medically vaporized herbal cannabis have previously been performed with a tabletop model, S&B Volcano, which has a greater capacity to evaporate cannabinoids due to the instrument design and the range of working temperatures [30]. Human pharmacokinetic information for handheld devices is not readily available and it can largely depend on cannabinoid extraction efficiency, which may vary between devices [15]. Therefore, we aimed at providing simple instructions for first-time users to quickly gain control over cannabinoid dosing and speed of onset while minimizing the risk of involuntary overdosing. However, it is worth noticing the relatively safe profile of the inhaled route compared to the sublingual or oral administration. Firstly, due to their lipophilic nature, sublingual absorption of cannabinoids in oily carriers is limited and almost identical to oral ingestion [55]. Secondly, several studies have reported that intoxication, acute psychiatric symptoms, and adverse cardiovascular events are more common in patients following oral ingestion of CBPMs, while hyperemesis syndrome (cycling vomiting) was more likely attributable to inhalation of herbal cannabis [56]. Finally, in response to the clinical requirement of prescribing THC-predominant cannabis flos for extended periods of time in patients experiencing benefit, clinicians should be aware of the relevant contraindications to this substance including psychotic vulnerability and cardiovascular instability, as well as the risks of patients developing cannabis use disorders (CUD).

This work presents several limitations, some of which are inherent to the way real-world data is collected and interpreted [57]. We used a convenience cohort which, while representative of the more than 3,000 patients enrolled by T21 over the last two years, still poses a high risk of selection bias [58]. Additionally, patients were grouped for analysis of PROMS by primary indication, but their diagnosis and etiology could differ. Although a dosing protocol was suggested, it is plausible to assume that each patient established their own individualize dosing regime and that some may not have used an herbal vaporizer to administer their CBPMs, which is also representative of real-world clinical practice [39]. Finally, our research design did not control for placebo effect, which is typically robust in studies using cannabis [58], although this could be partially mitigated by the flexible dosing regimen [10]. Owing to the inherent psychoactivity associated with the central activation of CB1 receptors, complete blinding in studies using THC is virtually impossible. In fact, relief of spontaneous pain typically correlates with high drug-like scores in human lab studies [18]. Therefore, aiming at completely separating the therapeutic properties from the psychoactive properties of THC may be erroneous, as some level of mind alteration may be required for the analgesic effect of cannabis to occur.

## 5. Conclusions

Our results indicate that controlled inhalation of pharmaceutical grade, THC-predominant cannabis flos was associated with a robust improvement in patient-reported pain scores, general mood, anxiety, sleep, and overall HRQoL in a treatment-resistant clinical population. The effect size, which was larger in patients diagnosed with anxiety disorders compared to chronic pain, appeared to be maximal at 3 months and sustained for at least 6 months. Occurrence of side effects was minimal, probably due to the previous experience of participants with cannabis inhalation. This evidence supports the notion that the administration of cannabis flos in a medicalized environment under the supervision of a trained healthcare provider further improves the clinical outcomes of legally prescribed CBMPs when compared to chronic patients self-medicating with illegal cannabis.

## Figures and Tables

**Table 1:** Cohort demographics, previous cannabis use and primary diagnosed conditions.

	Gender			Total
	Male	Female	Non-Binary	
Participants				
Sample: N (%)	267 (77.6)	76 (22.1)	1 (0.3)	344 (100)
Age: Mean ± SD	38.3 ± 10.6	38.6 ± 9.89	42 ± 0.0	38.4 ± 10.4
Previous Experience with Cannabis: N (%)	259 (97.0)	69 (90.8)	1 (100)	329 (95.6)
Intention of treating their primary condition with cannabis: N (%)	205 (76.8)	56 (73.7)	1 (100)	262 (76.1)
Frequency of cannabis use: N (%)				
Weekly	2 (0.75)	1 (1.32)	0	3 (0.87)
A few times a week	29 (10.9)	12 (15.8)	0	41 (11.9)
Once a day	160 (59.9)	41 (53.9)	1 (100)	202 (58.7)
Multiple times a day	15 (5.62)	3 (3.95)	0	18 (5.23)
Did not answer the question	61 (22.8)	19 (25.0)	0	80 (23.2)
Primary Condition: N (%)				
Chronic painful conditions	134 (50.2)	40 (52.6)	1 (100)	175 (50.8)
Anxiety-related disorders	74 (27.7)	13 (17.1)	0	87 (25.3)
ADHD	19 (7.12)	5 (6.58)	0	24 (6.98)
PTSD	12 (4.49)	9 (11.8)	0	21 (6.10)
Other Mental Health	14 (5.24)	4 (5.26)	0	18 (5.23)
Insomnia	8 (3.00)	2 (2.63)	0	10 (2.91)
Autism Spectrum Disorder	2 (0.75)	0	0	2 (0.58)
Epilepsy	0	1 (2.33)	0	1 (0.52)
Other	4 (1.50)	1 (1.32)	0	5 (1.45)

**Table 2:** Comparative analysis on the influence of the two main participants sub-populations by primary indication, chronic painful conditions, and anxiety-related disorders, over the general PROMS: HRQoL, mood/depression and sleep quality. * *p* < 0.05; ** *p* < 0.01.

	General Health EQ5D VAS Score			Health-Related QoL EQ5D 5-Dimensions SUM			Mood/Depression PHQ-9 Score			Quality of Sleep PSQI Score		
	Mean T = 0	Mean T = 3	Mean Diff	Mean T = 0	Mean T = 3	Mean Diff	Mean T = 0	Mean T = 3	Diff Mean	Mean T = 0	Mean T = 3	Mean Diff
Anxiety (N = 107)	57.10	69.55	12.4	10.07	8.31	1.76	13.72	6.58	7.14	12.31	9.16	3.15
Chronic Pain (N = 174)	47.35	54.33	6.98 *	14.24	12.94	1.30	12.78	9.42	3.36 **	13.51	10.79	2.72

## Data Availability

The data presented in this work are available on request from the corresponding author. The data are not publicly available due to privacy and ethical reasons.
